# Development of tele-lifestyle-based multidisciplinary survivorship program for gynecologic oncology practice

**DOI:** 10.1093/oncolo/oyaf033

**Published:** 2025-03-20

**Authors:** Nathalie D McKenzie, Nnamdi I Gwacham, Julie W Pepe, Sarfraz Ahmad, James E Kendrick, Robert W Holloway

**Affiliations:** AdventHealth Cancer Institute, Gynecologic Oncology Program, Orlando, FL 32804, United States; AdventHealth Cancer Institute, Gynecologic Oncology Program, Orlando, FL 32804, United States; AdventHealth Cancer Institute, Gynecologic Oncology Program, Orlando, FL 32804, United States; AdventHealth Cancer Institute, Gynecologic Oncology Program, Orlando, FL 32804, United States; AdventHealth Cancer Institute, Gynecologic Oncology Program, Orlando, FL 32804, United States; AdventHealth Cancer Institute, Gynecologic Oncology Program, Orlando, FL 32804, United States

**Keywords:** telemedicine, peri-habilitation, gynecologic cancer, survivorship, lifestyle, quality-of-life

## Abstract

We assessed the recruitment and retention of a short 8-week telemedicine-based group peri-habilitation program for gynecologic cancer survivors. Multidisciplinary team included: a gynecologic oncologist with additional board certification by the American College of Lifestyle Medicine, cancer-specific nutritionist, culinary medicine chef, physical therapist, exercise physiologists, mental health counselor, body image aesthetician, pelvic floor therapist, and sex therapist. Pre- and post-self-administered questionnaires assessed conformity to lifestyle medicine pillars and a general medical symptom questionnaire (MSQ). Recruitment was suboptimal (11.7%). Neither provider referrals nor flyers sufficiently directed patients to the program, but those that completed the program expressed meaningful impact on lifestyle behavioral change and improved quality-of-life across multiple parameters including MSQ (40.0 vs 20.75) and 85% participants reported compliance with recommendations. This pilot program suggests that a multidisciplinary tele-lifestyle-based survivorship program beyond just diet and exercise to improve quality-of-life in gynecologic cancer survivors, though novel and well received, needs physician buy-in and enhanced marketing strategies.

**Implications to Practice**

A multidisciplinary tele-lifestyle-based survivorship program is a viable strategy to improve quality-of-life in gynecologic cancer survivors but needs physician buy-in.

## Introduction

Gynecologic cancer survivors suffer from both physical and cognitive decline, while strategies to mitigate these and other quality-of-life (QoL) issues remain underutilized for this patient population.^1^ Yet, evidence suggests that adherence to the American Cancer Society nutrition and physical activity guidelines after a cancer diagnosis (ACS-NPG-AC) is associated with reduced morbidity, better QoL, and improved treatment outcomes.^2,3^ Indeed, the ACS-NPG-AC guidelines are consistent with recommendations published by other authoritative bodies including the American College of Lifestyle Medicine (ACLM) curriculum.^4^ Clinical interventions with diet or diet-plus-exercise are becoming more common, especially for other cancer types such as breast cancer.^5-7^ However, multimodality interventions incorporating all of the lifestyle pillars are largely absent and even more so for gynecologic cancers.^3,8,9^

Our tele-lifestyle-based peri-rehabilitation survivorship program (developed and modified during the COVID-19 pandemic) was branded as HEAL-GYN (Healthy Eating and Active Lifestyle—Gynecologic Cancer). This pilot program was developed to improve upon diet and exercise interventions for our gynecologic oncology patient population. HEAL-GYN focuses on all 6 pillars of lifestyle medicine.^3^ HEAL-GYN is led by a board-certified gynecologic oncologist with additional board certification by ACLM^4^ along with a multidisciplinary team who teach participants and lead activities on lifestyle pillars as recommended by the ACLM/ACS/AICR (American Institute for Cancer Research). Sexual dysfunction/intimacy/body image education is also provided.^3^ The specific interventions use a highly interactive educational format which incorporates motivational interviewing and goalsetting over 8-weekly consecutive shared peer-mentoring 90-minute sessions.^3^ During the pilot phase, we sought to explore recruitment strategies, feasibility, retention, behavioral impact, and short-term QoL for survivors.

## Methods

### Design

A longitudinal prospective pilot design with pre- and postquestionnaires using self-administered inventories about healthy behavior and a comprehensive medical symptom questionnaire (MSQ). All patients were interviewed after completing the program. We also used a team-based approach to conduct descriptive qualitative appraisal from focus group discussions, directly informed from the semi-structured interview questions that captured perceptions of acceptability of the program. Paired sample nonparametric tests were employed for the individual tests. No *P*-value adjustments were made for multiple tests conducted.

### Patient population

Persons with a diagnosis of any gynecologic cancer treated at a single gynecologic oncology practice consisting of 4 physicians and 3 mid-level providers over an 18-month period.

### Recruitment and program referral

Flyers were displayed on walls and doors within exam rooms, hallways, and as leaflets available at the practice check-out counter. Referral to the program relied on recall by physicians/providers during end of treatment discussions or cancer surveillance visits. Target patient population for the program was persons having completed treatment for gynecologic cancers within the previous 6-months; however, all patients were welcome to self-refer.

### Retention

In addition to the hospital systems automated 24- to 48-hour previsit call/text reminders, 30 minutes prior to each of the 8-sessions, a medical assistant called all registered participants to make an additional reminder and to identify electronic issues precluding participation in that day’s session. Reasons for not being able to or no longer wanting to participate in the program were noted.

### Pilot program description

Eight consecutive telemedicine-based shared medical visits each with a different focus topic. Our telemedicine team members included a cooking demonstration with a culinary medicine chef, a discussion with a registered dietician to discuss food quality, calories, and nutrient density, a mental health counselor, an exercise physiologist, and personal trainer instruction that included group exercise to provide safe alternatives to traditional exercise. Emphasis placed on the 6-pillars of lifestyle medicine^3,4^ (ie, whole-food plant-predominant diet, physical activity, sleep hygiene, stress management, “social connectedness,” limiting/avoid risky substances, eg, cigarettes). Prior to beginning the program, all participants received a printed copy of the ACLM adult-starter kit booklet highlighting strategies to enhance the 6-pillars. All 8-week cohorts received their program in the following sequence: Week 1: Intro and thorough discussion of all 6-pillars of lifestyle medicine ending with focus on social network optimization. Week 2: Healthy cooking demonstration inclusive of shopping and meal prep counseling. Week 3: Stress management. Week 4: Physical activity/exercise education and Yoga class participation. Week 5: Healthy eating for Cancer lecture and individual coaching. Week 6: Sleep optimization and strength training class. Week 7: Residual side effect education with body image and sexual issue lecture. Week 8: Conclusion and coaching for sustainability. Sessions concluded with each participant stating their SMART (Specific, Measurable, Attainable, Realistic, Time-specific) goal for that session’s topic. Each telemedicine encounter ranged from 90 to 120 minutes and were conducted every Wednesday in 8-consecutive week blocks. The physician billed for their time and the remainder of the health team’s time and supplies were covered through an intramural philanthropic grant. Participants were invited to join a closed-private social media group for peer-engagement and support. At the end of the feasibility study, additional resources were made available to provide individual consultations with a health coach beyond the program for the period of 1-year.

## Results

**Figure 1.**
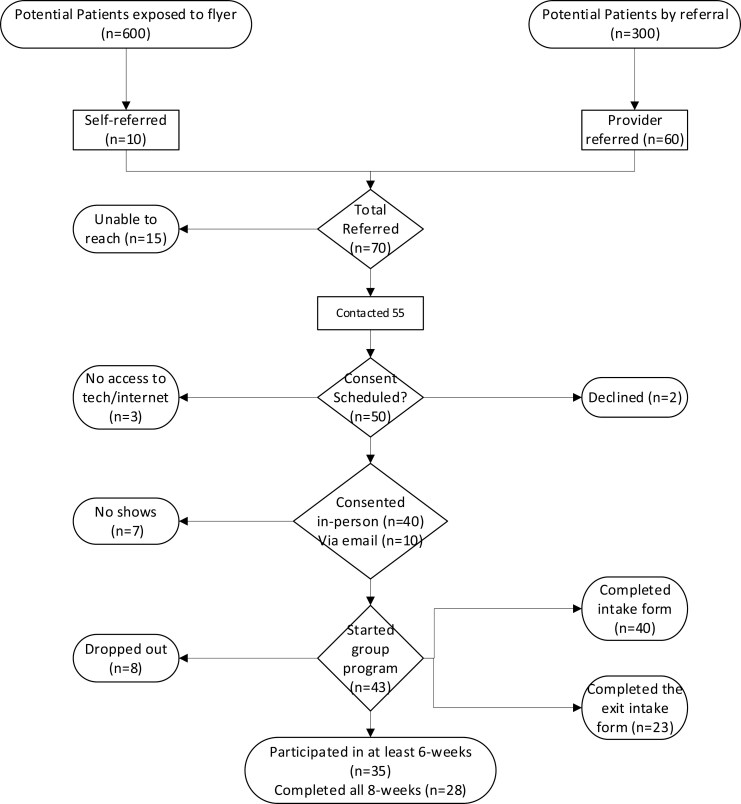
Recruitment and retention flow chart of the Healthy Eating and Active Lifestyle for Gynecologic Cancers pilot program.

Seventy gynecologic cancer patients were initially referred to this pilot program (12-month period) and 50 patients enrolled, of which 23 had complete data (Figure 1). Patients’ mean age was 58.8 years and mean starting body-mass index was 29.9 kg/m^2^. Patients with other (nongynecologic cancer types) who asked to join the program are not included in this report.

Participants reported a statistically significant improvement along several parameters, specifically, perceived stress, levels of anxiety, and depression decreased. Levels of purpose and social connection increased. Significant improvements were also noted in patients’ eating patterns and ability to manage their weight during the 8 weeks (Table 1). Only 4 participants completed the MSQ section of the survey; however, there was a notable decrease in general symptoms (Table 1). Patients reported an increase in perceived levels of overall health after completion of the program although small numbers preclude statistical significance.

**Table 1.** Results of multiscale lifestyle scores. Various domains of overall health reported by the ACLM self-administered inventory within the HEAL-GYN program.

	Pre-HEAL-GYN (±SD)	Post-HEAL-GYN (±SD)	*P*-value	*N* used
BMI (kg/m^2^)	29.13 ± 8.81	30.11 ± 8.36	**.046**	17
Sleep scores	31.86 ± 5.87	27.64 ± 5.28	**.001**	21
Medical symptom questionnaire	43.0 ± 23.02	20.75 ± 16.52	ND*	4
Overall health	5.94 ± 1.73	6.95 ± 1.66	.061	16
Eating patterns	4.96 ± 1.92	3.57 ± 1.59	**.001**	23
Weight management	9.78 ± 2.41	9.61 ± 1.95	.689	23
Perceived stress	11.48 ± 3.58	10.30 ± 3.27	**.002**	23
Resilience	29.26 ± 4.39	31.22 ± 4.31	**.014**	23
Anxiety/Depression	12.19 ± 8.68	6.91 ± 5.69	**.007**	21
Purpose/Connection	34.73 ± 4.0	37.0 ± 3.57	**.003**	22

*MSQ section (*n* = 4) showed drastic change; however, lower number limits meaningful statistical analysis. Abbreviations: MSQ: Medical Symptom Questionnaire; ACLM: American College of Lifestyle Medicine; HEAL-GYN: Healthy Eating and Active Lifestyle—Gynecologic Cancers; BMI: Body Mass Index.

The participants’ self-reported overall compliance with the varying elements of the HEAL-GYN program was generally very high (90%). Additionally, 100% of participants would “highly recommend the program” and none complained of stress or altered mood associated with online instructions.

Mixed-method qualitative open-ended survey [feedback for program improvement] by participants included the following suggestions: “better for patients who are done with treatment as sometimes I felt to weak or nauseous to participate fully,” “advertise this program more, because I would have joined sooner if I knew about it,” “I wish we could follow-up after the 8-week program was over,” “My job worked it out with me, but I wish this program was offered after working hours for people like me who work 9-5 jobs” and “I did see the flyers, but my own doctor didn’t bring it up, so I didn’t think it was important, maybe all doctors should recommend it.”

## Discussion

Ideal study design for multimodality lifestyle-based interventions remains a challenge and may vary depending on cancer site and individual patient functionality status. We report on the acceptability of a multimodality tele-lifestyle-based survivorship pilot intervention to inform a larger prospective study. Whether diet and exercise alone play the largest role in lifestyle-contributing outcomes remains unclear; however, the addition of other pillars of wellness is unanimously reported as very important by our participants. Limitations of our study include the relatively small numbers with complete data precluding our ability to make any claims regarding benefit. However, our results provide pilot data that a multidisciplinary peri-habilitation survivorship program for gynecologic cancer survivors using a telemedicine-based platform could be acceptable (100% of those who completed, “high recommended” the program), with ~81% retention (35/43 completed at least 6-sessions). Furthermore, this short 8-week program demonstrates the potential to positively impact patients reported multiscale lifestyle scores and physical function. Notably, we found suboptimal referrals (20%; 60/300 survivors seen in that time period) to this program from within our practice Therefore, education and “buy-in” from our colleagues is crucial to increase referrals. Additionally, 600 patient traffic in hallways exposed to flyers on walls but 10 “self-referred,” we suspect that increased funding aimed at patient-centered marketing of our program may be another opportunity to increase participation. Furthermore, it is important to highlight that interest in, compliance with, and referral to like programs are likely variable per institution, geography, resources, and beliefs.

## Data Availability

The data underlying this article will be shared on reasonable request to the corresponding author.
